# Quality of Primary Care for the Adult Population With Autism Spectrum Disorder: Protocol for a Scoping Review

**DOI:** 10.2196/28196

**Published:** 2021-11-19

**Authors:** Shannon Marion Aylward, Alison Farrell, Anna Walsh, Marshall Godwin, Roger Chafe, Shabnam Asghari

**Affiliations:** 1 Division of Clinical Epidemiology Faculty of Medicine Memorial University St. John's, NL Canada; 2 Health Sciences Library Faculty of Medicine Memorial University St. John's, NL Canada; 3 Discipline of Family Medicine Faculty of Medicine Memorial University St. John's, NL Canada; 4 Faculty of Medicine Memorial University St. John's, NL Canada; 5 Janeway Pediatric Research Unit Memorial University St. John's, NL Canada; 6 Division of Community Health Faculty of Medicine Memorial University St. John's, NL Canada

**Keywords:** autism spectrum disorder, primary care, family physician, quality, scoping review, protocol

## Abstract

**Background:**

A strong primary care system is vital to overall health. Research on the primary care of people with autism spectrum disorder (ASD) has mostly focused on children. A synthesis of the existing literature related to the quality of primary care for the adult population with ASD would elucidate what is known about the topic as well as inform future research and clinical practice.

**Objective:**

The purpose of our scoping review is to describe what is known about the quality of primary care for adults with ASD and identify knowledge gaps.

**Methods:**

Prior to beginning the literature search, we reviewed literature related to defining both primary care and primary care quality to establish the context and concept of the research question. The search strategy was designed and executed by a research librarian. The MEDLINE, CINAHL, EMBASE, PsycINFO, and ProQuest Dissertations and Theses databases were searched for relevant literature. Grey literature will include relevant reports from government websites and associations with a focus on ASD. Two members of the research team will independently screen the academic and grey literature. Quantitative, qualitative, or mixed methods study designs involving the quality of primary care services or patient-centered care for adults with ASD are eligible for inclusion in our scoping review. Studies that make it past the full-text review will undergo data extraction and quality appraisal by 2 independent reviewers. The data extraction results will be presented in a tabular format to clearly present what is known about the quality of primary care for adults with ASD; this table will be accompanied by a narrative synthesis. Literature selected for extraction will be coded for themes, which will form the basis of a thematic synthesis. The scoping review will follow the guidance proposed by the Joanna Briggs Institute.

**Results:**

The search of electronic databases was conducted in October 2020, and it returned 2820 results. This research is still in progress. The results from our scoping review are expected to be available by fall 2021.

**Conclusions:**

The results from our scoping review will be useful for guiding future research on the quality of primary care for adults with ASD.

**International Registered Report Identifier (IRRID):**

PRR1-10.2196/28196

## Introduction

### Background

Autism spectrum disorder (ASD) was first described in 1943 by Kanner [1]. In terms of presentation, ASD symptoms fall along a spectrum but often involve deficits in reciprocal social interaction and communication as well as the presence of restrictive and repetitive behaviors [2,3]. The global prevalence of ASD is estimated to be 1 in 160 persons [4]. It has been noted that people with ASD have poorer health outcomes and use the health care system differently from the general population [5,6]. Primary care providers are often an individual’s first point of contact with the health care system, and they facilitate access to additional health care services [7]. The majority of ASD research has focused on children, and as such, most primary care research related to ASD occurs in a pediatric care setting. There is little known about how ASD in adulthood is managed within the adult primary care system. This represents a significant gap in the literature, as people with ASD have a high number of physical and psychiatric comorbidities, which may worsen as they continue to age [5]. The existing literature on the primary care of adults with ASD must be examined to guide future research in this field.

There has been much research on defining the attributes of primary care and the quality of care [7-9]. One framework suggests that there are 2 main measures of quality—accessibility and effectiveness [7]. Under this framework, effectiveness is broken down into the effectiveness of clinical care and patient-centeredness [7]. Similarly, one systematic review indicated that patient-centeredness and the quality of the following primary care services are indicative of primary care quality: the prescribing behaviors of primary care providers, diagnosis and treatment in primary care, the management of chronic diseases, mental health care, maternal and child health care, preventive care, and health promotion [8]. Patient-centered care is “an approach to practice established through the formation and fostering of healthful relationships between all care providers, service users and others significant to them in their lives…underpinned by values of respect for persons, individual right to self-determination, mutual respect and understanding” [10]. The focus of these frameworks on clinical care and patient-centered care makes them appropriate for the study of ASD due to the medical complexities and interpersonal deficits associated with ASD.

The objective of our scoping review is to investigate the evidence related to the quality of primary care for adults with ASD. The study will review literature involving an assessment of primary care quality for adults with ASD, including assessments of the quality of the clinical care provided as well as the quality of interpersonal interactions among care providers, patients, and patients’ families [7,11]. This conceptualization will guide the screening and extraction of the literature. As indicated by the Joanna Briggs Institute, scoping reviews are an appropriate approach for synthesizing existing evidence and identifying gaps in available knowledge [12]. Furthermore, a preliminary literature search returned few literature results. This undermined the feasibility of a systematic review.

As ASD is a lifelong condition, individuals diagnosed with ASD will inevitably encounter the adult primary care system. The core features of ASD include difficulties with social interaction and communication, which can complicate the receipt of medical care. Physicians’ lack of understanding of ASD, as well as poor communication with health care providers, presents barriers to health care for adults with ASD [13]. This review may shed light on whether the current adult primary care system is meeting the needs of adults with ASD, which in turn may inspire the design and evaluation of appropriate care models.

### Review Questions

Our main research question is as follows: what is known about the quality of primary care for the adult population (aged ≥18 years) with a diagnosis of ASD receiving care in the adult primary care system? The following are our secondary research questions: (1) what types of patient-centered health or health service measures have been reported in the literature related to the primary care of adults with ASD and (2) what are the evidence gaps related to the quality of primary care for adults with ASD?

## Methods

This paper describes a scoping review protocol that follows the guidance proposed by the Joanna Briggs Institute [12].

### Inclusion Criteria

The population, concept, and context approach was used to guide the construction of the protocol inclusion criteria [12].

### Participants

Our scoping review will include studies involving individuals with ASD who are over the age of 18 years. In terms of presentation, autism symptoms fall along a spectrum but often involve deficits in reciprocal social interaction and communication as well as the presence of restrictive and repetitive behaviors; symptoms must be present “in the early developmental period” [2,3]. To optimize the sensitivity of the search strategy and ensure that all articles related to ASD are included in our study, the search strategy was not restricted to articles involving individuals with a formal diagnosis of ASD. The ASD definitions that were identified in each individual study were used to guide the literature screening and extraction process.

### Concept

All studies that include an assessment of primary care quality will be included in the scoping review. To assess primary care quality, we will include any literature assessing patient-centered care as well as the quality of primary care services (ie, prescribing behavior, diagnosis and treatment, the management of chronic diseases, mental health care, maternal and child health care, health promotion, and preventive care) [8].

Patient-centered care is a complex concept. This scoping review will use a broad definition of patient-centered care and include all studies examining concepts related to patients’ relationships with care providers, patient and family involvement, and contexts in which care is delivered [10,11,14]. Literature with structured or unstructured measures of patient-centered care will be included.

### Context

The Institute of Medicine defines primary care as “the provision of integrated, accessible health care services by clinicians who are accountable for addressing a large majority of personal health care needs, developing a sustained partnership with patients, and practicing in the context of family and community” [15]. In this definition, a clinician “has direct contact with patients and may be a physician, nurse practitioner, or physician assistant” [15]. For the purposes of this review, included studies must indicate that the main provider of primary care is a family physician, general practitioner, internist, or advanced practice nurse (eg, a nurse practitioner) [16]. This scoping review will also include solo and group practice models, provided that the primary responsible provider is one of the provider types listed above. According to the Institute of Medicine, primary care can occur in a variety of health care settings (eg, clinicians’ offices, nursing homes, schools, etc) [15]. The scoping review will include studies in which primary care was delivered in a clinic setting (eg, a walk-in clinic) or during a home visit. Although the population and context of interest for this scoping review include young adults with ASD who are transitioning to adult primary care, studies specific to the process of transitioning from pediatric care to adult care will be excluded.

### Types of Studies

Our scoping review will consider quantitative, qualitative, and mixed methods study designs for inclusion. Original journal articles, including doctorate theses, as well as grey literature will be considered in this scoping review. Editorials, opinion papers, conference abstracts, and policy analyses will be excluded. There will be no restrictions on the language or year of publication during the literature search. All abstracts will be considered for a full-text review. If an article that is considered for the full-text review is not available in English, then a translation will be requested.

### Search Methods

#### Search Strategy

The search strategy for the retrieval of studies from electronic bibliographic databases and grey literature sources was designed by one of the authors (AF)—a research librarian. The initial search will involve a keyword search of MEDLINE (via Ovid). The titles and abstracts of retrieved articles will be reviewed to identify appropriate keywords and index terms. The final search will be designed in MEDLINE (via Ovid), translated, and conducted in select databases. Table 1 presents the full MEDLINE search strategy. Backward and forward citation searching will be performed on articles that are selected for data extraction. If required, the authors of selected studies will be contacted to obtain full articles or further information.

**Table 1:** Ovid MEDLINE search strategy.^a^

Search number	Search strings	Number of studies found
1	*Primary Health Care*	78,551
2	*Physicians/ or Physician Assistants*	95,184
3	*exp General Practice/ or exp General Practitioners*	81,530
4	*exp Primary Care Nursing*	496
5	*office visits/ or community mental health services/ or ambulatory care/ or community health services/ or community health nursing/ or community medicine*	118,668
6	*((primary or basic) adj2 (healthcare or care)).tw,kf*	150,228
7	*((general or family) adj (physician or physicians or doctor or doctors or practitioner or practitioners or practice)).tw,kf.*	105,743
8	*family medicine.tw,kf.*	11,093
9	*((physician or doctor or physicians or doctors) adj (assistant or assistants or extender or extenders)).tw,kf.*	4590
10	*(internist or internists).tw,kf.*	7311
11	*nurse practitioners/ or family nurse practitioners/*	17,764
12	*nurse specialists/ or nurse clinicians/*	8429
13	(nurse adj (specialist* or practitioner* or clinician*)).tw,kf.	16,172
14	*walk in clinic.tw,kf*	265
15	*advanced practice registered nurse.tw,kf*	193
16	*first contact.tw,kf.*	2522
17	*exp child development disorders, pervasive/*	36,166
18	(autis* or asperger* or ASD or pervasive development disorder*).tw,kf.	57,925
19	17 *or* 18	61,672
20	1 *or* 2 *or* 3 *or* 4 *or* 5 *or* 6 *or* 7 *or* 8 *or* 9 *or* 10 *or* 11 *or* 12 *or* 13 *or* 14 *or* 15 *or* 16	507,395
21	19 *and* 20	820

^a^The date of search was October 22, 2020.

#### Information Sources

The databases that will be searched will include MEDLINE (via Ovid), CINAHL (via EBSCO), EMBASE (via Embase.com), PsycINFO (via EBSCO), and ProQuest Dissertations and Theses. The search for grey literature will include searching for reports on the websites of departments and ministries of health and associations with a focus on ASD. This will help us to identify reports related to primary care for adults with ASD.

#### Study Selection

After the completion of the literature search, the research librarian will remove any duplicate citations and upload the remaining citations into Covidence—a web-based software program for managing literature reviews [17]. By using Covidence, 2 team members (SMA and AW) will independently assess the results of the published and grey literature searches. The same screening criteria will be used for all types of included evidence. In the first phase, the titles and abstracts of all records will be assessed against the following screening questions: (1) was a quantitative, qualitative, or mixed methods study design used; (2) do the study’s participants involve or reference adults with ASD; (3) does the study involve the receipt of health care from a primary care clinician (family physician, general practitioner, internist, or advanced practice nurse) in a primary care setting (clinic or home visit); and (4) is there an assessment of the quality of clinical services or patient-centered care? To maximize the inclusion of relevant studies, a title or abstract that appears to meet all screening criteria but lacks sufficient detail for fully assessing a study’s eligibility will move forward to the full-text review. All discrepancies about whether a study meets the inclusion criteria will be resolved by consensus; in cases where a consensus cannot be reached, a third team member will be consulted.

Prior to the initial screening phase, the screening questions will be pilot-tested by the two reviewers using a sample of 50 articles retrieved from the MEDLINE (Ovid) database. The team members will meet weekly to discuss discrepancies and make modifications to the eligibility criteria and definitions guiding the review. Once ≥75% agreement is achieved, the initial phase of screening will commence [12].

The next phase of screening will involve reviewing the full texts of all retained citations by using the same screening questions. Disagreements about study inclusion will be resolved by a third team member (SA). The reasons for exclusion will be documented. The results of the search and screening will be reported in the final scoping review and presented in a PRISMA (Preferred Reporting Items for Systematic Reviews and Meta-Analyses) flow diagram [18].

Although scoping reviews do not generally include an appraisal of the quality of included literature [12,18], studies that are included in our scoping review will undergo critical appraisal. A quality appraisal will add another layer to the findings related to research gaps in the primary care of adults with ASD and thus better direct future research endeavors. Included studies will be appraised by using the 2018 version of the Mixed Methods Appraisal Tool [19]—a reliable tool that can be applied to the appraisal of quantitative, qualitative, and mixed methods study designs [19-21].

### Data Extraction

Two reviewers will use a tool designed by the study team to independently extract information from sources based on predefined criteria. The tool is displayed in Multimedia Appendix 1. The effectiveness of clinical care and the effectiveness of patient-centered care are the aspects of quality that will be extracted. Following the extraction of data from the first 2 included sources, the two reviewers will meet to determine whether the tool extracts all relevant information and modify the tool as necessary before resuming the review and extraction of the remainder of the sources [12].

### Data Analysis and Presentation

We will evaluate the number of studies related to the quality of primary care for adults with ASD. We will describe the trends in the studied sample, the study designs, the aspects of quality that were measured, and the methods of quality assessment. The results of the scoping review will be presented in a table, which will be accompanied by a narrative synthesis that will connect the results to the research objectives of the scoping review [12]. The narrative synthesis will involve a summary and description of the findings of the extracted studies. Our scoping review will use a data-based convergent synthesis design whereby all included studies will be analyzed by using a qualitative approach (ie, thematic synthesis) [22]. If required, data transformation will be performed. The analysis will be carried out by one author (SMA) and validated by another author (AW). Qualitative data analysis software will be used to code the included literature for themes. These themes will form the basis of the qualitative thematic synthesis. The results will be reported by using the PRISMA scoping review reporting guidelines [18].

## Results

The search of electronic databases was conducted in October 2020. A total of 2820 results were retrieved, of which 34 were identified as duplicates. Our scoping review was in the full-text screening phase in February 2021. Data extraction and thematic synthesis will occur during the summer of 2021. The completed scoping review is expected to be submitted for publication in December 2021.

## Discussion

Our scoping review will examine the available evidence related to the quality of primary care for adults with ASD and seek to identify gaps in existing knowledge. The scoping review protocol presented in this paper is novel; to our knowledge, no review has been conducted on the quality of primary care services for the adult population with ASD [23-25]. Reviews that focus on health care for adults with ASD have been specific to access to health care and not to the quality of the services received [13,26,27]. This scoping review will identify gaps in the literature and provide insight into future research needs related to the provision of quality primary care for adults with ASD.

## Appendix

Multimedia Appendix 1 Data extraction form.

## Abbreviations

ASD: autism spectrum disorder
PRISMA: Preferred Reporting Items for Systematic Reviews and Meta-Analyses
