# Supplementary material for: Quality of Primary Care for the Adult Population With Autism Spectrum Disorder: Protocol for a Scoping Review
Source: JMIR Res Protoc. 2021 Nov 19;10(11):e28196. doi: 10.2196/28196 (PMC8663610; doi:10.2196/28196)
Supplement: Multimedia Appendix 1 [file resprot_v10i11e28196_app1.docx]

# Appendix 1: Data Extraction Tool

| **Scoping Review Details** | |
| --- | --- |
| Scoping Review title: | Title: Quality of primary care for the adult population with autism spectrum disorder (ASD) |
| Review objective/s: | Review Objective: The objective of this scoping review is to investigate the evidence related to the quality of primary care for adults with ASD. |
| Review question/s: | Review Questions: What is known about the quality of primary care for the adult population (age ≥ 18 years old) with a diagnosis of ASD in receipt of adult primary care services? i. What types of patient-centred, health or health service measures have been reported in the literature relating to the primary care of adults with ASD? ii. What are the evidence gaps related to the quality of primary care for adults with ASD? |
| **Citation Details** | |
| First Author's name and contact information (e.g. university affiliation) |  |
| Article Title |  |
| Journal Name |  |
| Year Published |  |
| Country |  |
| **Study Characteristics** | |
| Study Objectives |  |
| Context |  |
| Type(s) of Care Providers |  |
| Inclusion Criteria |  |
| Exclusion Criteria |  |
| Number of Participants |  |
| Description of Participants (e.g. age, sex, relationship to a person with ASD, relevant diagnoses etc.) |  |
| **Method** | |
| Study design |  |
| Recruitment strategy |  |
| Interventions (if applicable) |  |
| **Details/Results extracted from source of evidence** | |
| Broad Concept (Quality of care domain assessed) |  |
| Specific measures of quality |  |
| Methods/tools used to measure quality |  |
| Outcome(s) |  |
| Notes |  |
